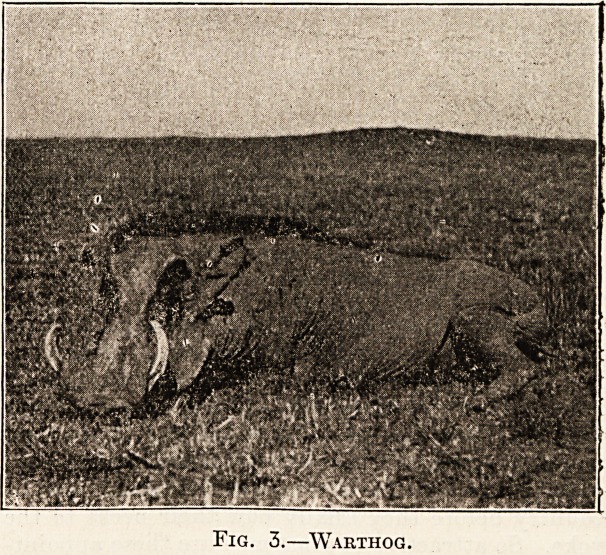# Travel and Big Game Shooting

**Published:** 1907-06-01

**Authors:** 


### The Practitioner's Relaxations and Hobbies.

### TRAVEL AND BIG GAME SHOOTING.

When members of the other learned professions discuss the advantages and disadvantages of medicine as a career, they frequently dilate upon the facility with which a young medical man may " see the world " at little expense, or even on terms of actual remuneration. Nor is there any doubt that such chances do occur in our profession much more often than in others. There must be a great number of practitioners who have fulfilled the duties of a ship's surgeon for one or more voyages to distant shores. Pleasanter or healthier relaxation could not be desired than a long passage on a big liner, especially after the weary toil of examinations or the enervating fatigue of house office. Unlike the barrister or the public schoolmaster, the medical man, when once engaged in practice, is often confined for life to a village or small country town, with holidays so brief as to preclude any travel further than Cisalpine Europe. It is not strange that young and active men with such expectations should accept eagerly the chance of voyaging to some distant country before they finally bow their necks to the yoke. So attractive to some men are these appointments that the scanty pay and lack of prospects are not able to detach them from the sea; in the employ of every big line there are surgeons who have passed the best years of their lives at sea, and they are, as a rule, placed in charge of the largest and most modern ships.

Fig. 1.—Camp, Rongai River, British East Africa.

More lucrative, and even more comfortable, are the posts on private ocean-going steam yachts, but they are, of course, distinctly rare. There is no more halcyon an existence than that of the yachtsman aboard one of these palaces of luxury. The house party and their surgeon cruise from port to port or from island to island, and it is especially in the least frequented and least hackneyed places that they are most warmly welcomed by the inhabitants. There is no immutable route marked out, no strict time schedule to be observed, and the length of stay in any port is determined solely by the time required to exhaust its attractions. Moreover, a yacht generally visits a much larger number of places than her big sisters—the liners. Some yachtsmen seek opportunities for sport, and their surgeons have been known to try their luck at turtle turning, shark catching, tarpon fishing, wild-fowling, and other exciting pursuits.

The foreign travels incidental to the medical services of the Navy and the Army are perhaps not strictly recreative, but part and parcel of duty; they are at least a serious inducement in the eyes of many of the candidates for admission. A few years ago the late Boer war provided for many young practitioners the well-paid and usually pleasant duties of civil surgeons, with the added attractions of medals and glory. Arctic and Antarctic expeditions have before now offered chances of distinction to their medical officers, who have frequently been men of varied talents—artistic, geological, or biological, as well as medical. The medical charge of semi-invalid or convalescent patients are often better paid, and a continental tour of this kind may be extremely pleasant. In the Colonial medical services, particularly those in tropical Africa, the routine of professional work is often disturbed in many ways. Medical men have been known to act as commander-in-chief, judge, sheriff, executioner, tax collector, and what not, as well as P.M.O., in outposts of the empire where but one white official can be allotted to each station. They often find compensatory recreation in the most thrilling, because the most dangerous, sport in the world—big game shooting. But the surgeon, whose chances of this are really unique, is he who travels with a private shooting party. With improved roads and the building of railways the need for a medical man is much reduced, and many parties now go without one; but the big game hunter can never be secure from the risks of serious injuries

Fig. 2.—Grant's Gazelle.

and of tropical fevers, and prompt surgery may save a limb or a life necessarily sacrificed when help is hundreds of miles away.

### On the East African Veld.

In healthy countries, such as the highlands of East Africa, British Central Africa, and Abyssinia, life on "safari" is ideal. By dawn the camp is astir, and before sunrise the white man too is out of his blankets. While he attacks breakfast his boy packs the camp bed and other baggage, and the porters fold up his tent. The latter pick up their 60 lb. loads, their master and his gunbearer take the lead, and the procession winds in single file across spreading plains or over lofty mountains. Presently the laden porters are left behind, and coming cautiously to the brow of a slight rise the hunter sweeps the next fold from behind a bush. In front may be two or three large herds of one of the commoner species of antelope, but they are not wanted to-day; away to one side, perhaps, a crowd of lovely zebra, easy to stalk but difficult to kill. Then the sweep checks as something really worth chasing comes into the field of the glass; it may be a rhinoceros waddling about in his peculiarly aimless fashion, a herd of eland with a fine bull, a kudu, a waterbuck, or a sable antelope, say. Word is sent to the caravan to make a detour out of sight, and to pitch camp by a distant hill where the guide says there is a stream, and the stalk begins. Where or when it may end is on the knees of the gods; often the most cunning shikari is forced to abandon his stalk miles from his route perhaps to start a more successful one as he is making for the rendezvous. As he seeks this he must note the landmarks by which to direct the subsequent recovery of the prize, and keep a careful watch for the trail of his porters. A huge meal in camp and a lounge in the tent pass the time until some 40 minutes before sunset, when a short excursion for game birds, and the off-chance of a prowling lion, concludes the labours of the day. A typical camp includes large square double-walled "explorer's" tents of the type shown in Fig. 1, of which one is carried for each sportsman, and a number of small thin canvas shelters wherein the porters sleep huddled together for warmth at night. The latter are recruited almost exclusively from Bantu tribes, chiefly Swahili, Nyamwezi, Ukamba, or Kikuyu. The three former tribes carry their loads upon their heads, but the latter prefer to sustain them somewhere in the small of the back by a broad strap passing round the forehead. In consequence, they must bend continually forwards, and their gait is highly ungainly compared with the upright bearing of the coast men; but nevertheless they make excellent porters. The Nilotic peoples are averse to any form of work, especially the most intelligent and bravest tribe, the Masai, who do nothing at all! The study of these interesting natives is not the least of the fascinations of Equatorial Africa, for often a march of a few miles brings the traveller to a region where language, customs, weapons, and even animals are entirely different from those he has just left. Thus Grant's gazelle, Fig. 2, is found in two distinct forms in the highlands, and in two more near the coast. The illustration of this beautiful antelope is of the northern highland variety. The subject of Fig. 3, the warthog, is of a somewhat different order of beauty; though not so game as his Indian cousin; the warthog is quite capable of turning to bay if he is cut off from his earth, and a big tusker is well worth sticking. Sometimes a stay of several

Fig. 3.—Warthog.

days in one camp is made, and night watches for the king of beasts, or weary tramps after elephant or buffalo, are undertaken; sometimes the camp is moved daily. In either case the pleasures and fatigues of the chase, the observation and perhaps photography of wild nature, the free life, and keen breezes of the highland plateaux combine to render such expeditions delightful.

## Figures and Tables

**Fig. 1. f1:**
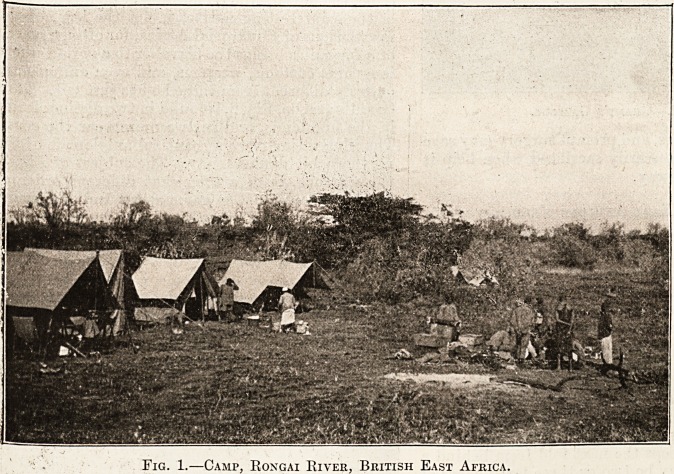


**Fig. 2. f2:**
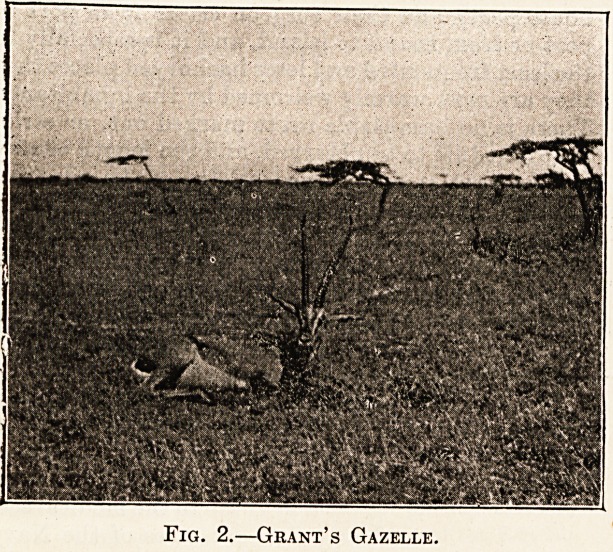


**Fig. 3. f3:**